# Loss of serum response factor induces microRNA-mediated apoptosis in intestinal smooth muscle cells

**DOI:** 10.1038/cddis.2015.353

**Published:** 2015-12-03

**Authors:** C Park, M Y Lee, O J Slivano, P J Park, S Ha, R M Berent, R Fuchs, N C Collins, T J Yu, H Syn, J K Park, K Horiguchi, J M Miano, K M Sanders, S Ro

**Affiliations:** 1 Department of Physiology and Cell Biology, University of Nevada School of Medicine, Reno, NV, USA; 2 Department of Physiology, Wonkwang Digestive Disease Research Institute and Institute of Wonkwang Medical Science, School of Medicine, Wonkwang University, Iksan, Jeollabuk-do, Korea; 3 Aab Cardiovascular Research Institute, University of Rochester School of Medicine and Dentistry, Rochester, NY, USA; 4 Division of Biological Science, Wonkwang University, Iksan, Jeollabuk-do, Korea; 5 Department of Anatomy, University of Fukui Faculty of Medical Sciences, Matsuoka, Fukui, Japan

## Abstract

Serum response factor (SRF) is a transcription factor known to mediate phenotypic plasticity in smooth muscle cells (SMCs). Despite the critical role of this protein in mediating intestinal injury response, little is known about the mechanism through which SRF alters SMC behavior. Here, we provide compelling evidence for the involvement of SRF-dependent microRNAs (miRNAs) in the regulation of SMC apoptosis. We generated SMC-restricted *Srf* inducible knockout (KO) mice and observed both severe degeneration of SMCs and a significant decrease in the expression of apoptosis-associated miRNAs. The absence of these miRNAs was associated with overexpression of apoptotic proteins, and we observed a high level of SMC death and myopathy in the intestinal muscle layers. These data provide a compelling new model that implicates SMC degeneration via anti-apoptotic miRNA deficiency caused by lack of SRF in gastrointestinal motility disorders.

Serum response factor (SRF) is a muscle-specific transcriptional factor that drives smooth muscle cell (SMC)-specific gene expression, which is necessary for contractile and cytoskeletal functions.^1, 2, 3^ SRF transcriptionally activates expression of SMC-specific genes by binding to the CArG box sequence [CC (A/T)_6_ GG] in promoters and introns of most SMC-restricted genes.^1^ Computational analysis of genome-wide CArG boxes (CArGome) in mice and humans has identified many SRF-dependent genes that encode for cytoskeletal/contractile or adhesion proteins, suggesting that SRF is a master regulator of the actin cytoskeleton.^4^ In addition, myocardin (MYOCD)-related transcription factors (MRTFs), which include MYOCD, MRTF-A/MKL1/MAL and MRTF-B/MKL2, directly interact with SRF to activate a subset of SRF-modulated genes to promote myogenic differentiation and cytoskeletal structuring.^3^ Although SRF is known primarily as the critical switch for induction of the muscle phenotype, it has recently been implicated in more diverse and multifunctional roles. For instance, there is now evidence that SRF is involved in carcinogenesis and tumor progression, induction of epithelial-to-mesenchymal transition with drug resistance in hepatocellular carcinoma and in lung fibrosis.^5, 6^ Furthermore, MYOCD, which is an integral part of the SRF/MRTF pathway, has also been recently implicated in apoptosis and autophagy of SMCs,^7^ and SRF has been shown to attenuate Myc-induced apoptosis in mammary epithelial cells in culture.^8^ However, as of yet, there has been no demonstration of apoptotic induction with knockout (KO) of *Srf* in SMCs.

MicroRNAs (miRNAs) are critical for SMC growth, differentiation and survival within the gastrointestinal (GI) track.^9^ Furthermore, several hundred miRNAs that determine cellular fate and phenotype, including SMC-specific miR-143 and miR-145, are expressed in SMCs.^10^ Depletion of Dicer, a RNase III that generates mature miRNAs, in mouse SMCs results in the degeneration of smooth muscle and severe myopathy within the GI tract,^9^ and a similar phenotype results with *Srf* ablation in mouse SMCs.^11, 12^ The cellular mechanism of SMC loss in the *Srf* KO mice, however, remains poorly understood.

We report here a model that describes the functional role of SRF in suppression of apoptotic activity through SRF-induced miRNAs that target apoptotic proteins in SMCs. Our proposed model reveals how loss of SRF expression can lead to SMC death and intestinal myopathy in the development of GI motility disorders.

## Results

### Conditional deletion of *Srf* in adult SMCs results in the severe dilation of GI tract

**Figure 1:**
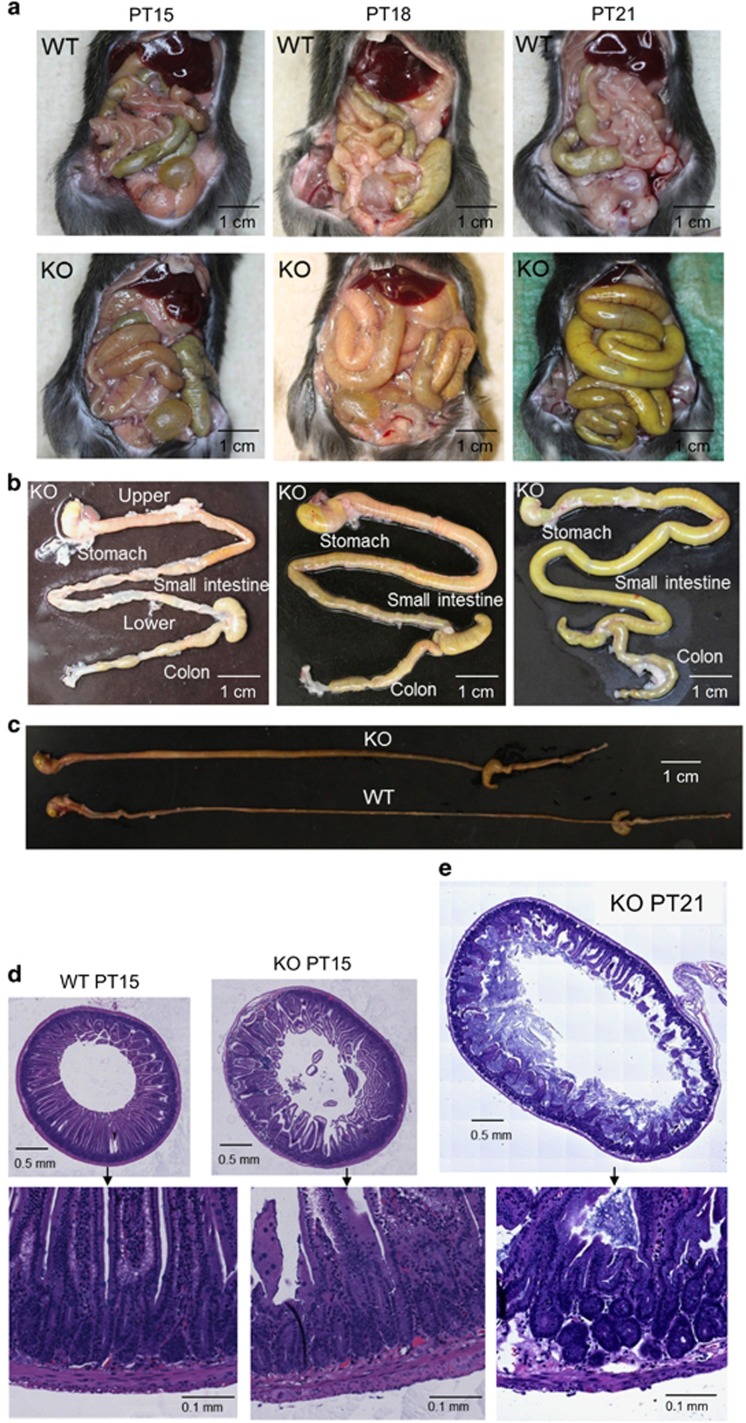
Smooth muscle defect of the inducible SMC-specific *Srf* KO in GI track. (**a**) Gross changes of GI tract between WT and KO at 15, 18 and 21 days post-tamoxifen injection (PT15, PT18 and PT21). (**b**) Morphological changes of *Srf* KO GI tract showing dilation of upper duodenum at PT15 progressing to lower GI tract at PT18–PT21. (**c**) Comparison of GI length between WT and KO. (**d**) Cross-sectional images of WT and KO jejunum with H&E staining at PT15. (**e**) Cross-sectional images of KO jejunum with H&E staining at PT21

Congenital *Srf* KO mice did not survive the prenatal stage.^13^ Therefore, an inducible SMC-specific *Srf* KO mouse line was generated. Following tamoxifen-induced KO, these adult mice developed progressive dilation and inflammation of the GI tract, which started in the upper duodenum as early as 15 days post-tamoxifen injection (PT15; Figure 1a). The dilation and inflammation later progressed to the lower GI tract (Figure 1b), and the severe dilation of the entire GI tract lead to death of affected mice between PT22–PT28. At the terminal stage (PT21), the small intestine was completely filled with watery chyme, whereas the colon was packed with hard feces (Figures 1a and b). Furthermore, the length of the GI tract from stomach to colon in the KO mice was significantly shorter than that of wild-type (WT) mice (Figure 1c). Cross-sectional images of KO jejunum revealed hyperplastic and hypertrophic growth of the smooth muscle at PT15 (Figure 1d) in addition to severe circumferential dilation with thinning of the smooth muscle layers at PT21 (Figure 1e).

### Cellular and phenotypic changes of KO SMCs

**Figure 2:**
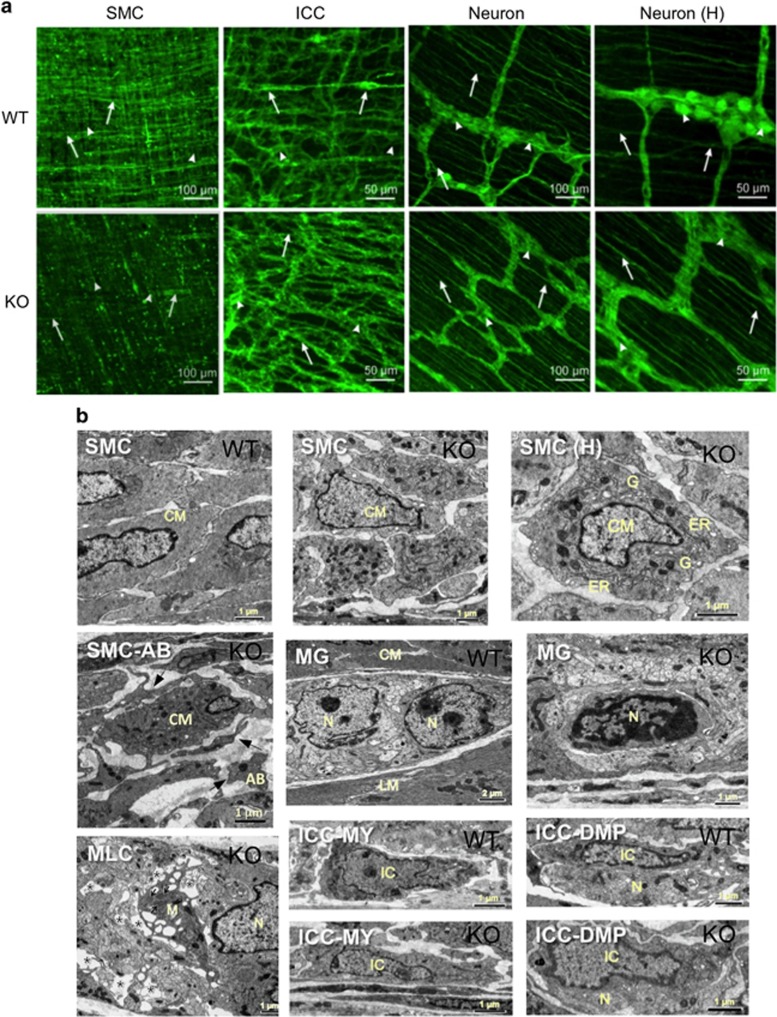
Cellular changes in *Srf* KO smooth muscle. Cellular changes in jejunal smooth muscle at PT21 were examined with cell marker antibodies using confocal or electron microscopy. (**a**) Whole-mount images showing phenotypic changes of KO SMCs compared with WT SMCs. SMCs were labeled with anti-MYH11 antibodies. Arrowheads and arrows indicate longitudinal and circular SMCs, respectively. Note structural defect in KO SMCs. Whole-mount images of ICC network. ICCs were labeled with anti-KIT antibodies. Arrowheads and arrows indicate ICC-MY and ICC-DMP, respectively. Whole-mount images of neuronal ganglia that were labeled with anti-PGP9.5 antibodies. Arrowheads and arrows indicate neuronal cells in ganglia and exons running in the muscle, respectively. High-magnification images (H) of neuronal cells are shown. Note significant neuronal cells were lost in KO ganglia. (**b**) Ultrastructural changes of KO and WT circular SMCs (CM). WT SMCs show normal ultrastructural features. The contour of KO SMCs is irregular and the extracellular space between the SMCs is wider than WT SMCs. A high magnification (H) of an irregular KO SMC is shown. A cellular fragmentation of a KO SMC surrounded by apoptotic bodies (SMC-AB: arrows). Ultrastructures of a MY ganglion (MG) containing neuronal cells (N) between the circular and longitudinal muscle layers in WT and KO muscle are shown. MLCs are observed within the ganglia. Arrows indicate lysosomes in the MLC (M). Asterisks show the damaged area within the ganglion. Ultrastructures of ICC-MY and ICC-DMP in WT and KO are shown. ICC-MY (IC) are located between circular and longitudinal smooth muscle layers and characterized by electron-dense cytoplasm and many mitochondria. ICC-DMP (IC) are closely associated with nerve bundles (N)

At PT21, SMCs in both circular and longitudinal muscle layers of *Srf* KO mice were significantly more disorganized with weaker expression of MYH11 compare with WT controls (Figure 2a). Notably, populations of SMCs in KO mice were abnormally enlarged and hypertrophic. In contrast, there was no significant qualitative or quantitative difference in the populations of myenteric (ICC-MY) and deep muscular plexus (ICC-DMP) interstitial cells of Cajal (ICCs) in WT and KO *Srf* mice as evidenced by anti-KIT staining (Figure 2a). However, anti-PGP9.5 staining of neurons revealed abnormal myenteric (MY) ganglia in KO jejunum compared with WT (Figure 2a). Specifically, *Srf* KO neuronal ganglia exhibited less cell bodies and more densely packed axons. Collectively, our data suggest that deficiency of *Srf* resulted in loss of contractile proteins, which may lead to morphological changes of SMCs and MY neurons.

Ultrastructural examination of SMCs with electron microscopy revealed that *Srf* KO SMCs had normal well-developed contractile filaments at PT5, PT10 and PT15. However, at PT21, KO SMCs began to display irregular contours and processes compared with age-matched WT controls (Figure 2b). Furthermore, the KO SMCs displayed well-developed rough endoplasmic reticulum and Golgi apparatus; widely distributed organelles and mitochondria; and less conspicuous contractile elements that were suggestive of an active synthesis state (Figure 2b). The intercellular spaces of KO muscular tissue, which contained numerous apoptotic cells, were also markedly wider when compared with WT muscles (Figure 2b). There was, however, no evidence of necrosis in KO muscles such as cell swelling, plasma membrane breakdown or organelle leakage.

Ultrastructural analysis of *Srf* KO GI tissue also revealed degenerative changes within the MY ganglia (MG) with macrophage-like cells (MLCs; Figure 2b) located around damaged ganglia. In contrast to the degenerated SMCs and neurons in *Srf* KO intestinal tissue, the ICC-MY and ICC-DMP appeared relatively normal in *Srf* KO muscles at least up until PT21 (Figure 2b). Collectively, the ultrastructural data confirmed the apoptotic activity of SMCs and degeneration of neurons in the intestinal ganglia of *Srf* KO mice.

**Figure 3:**
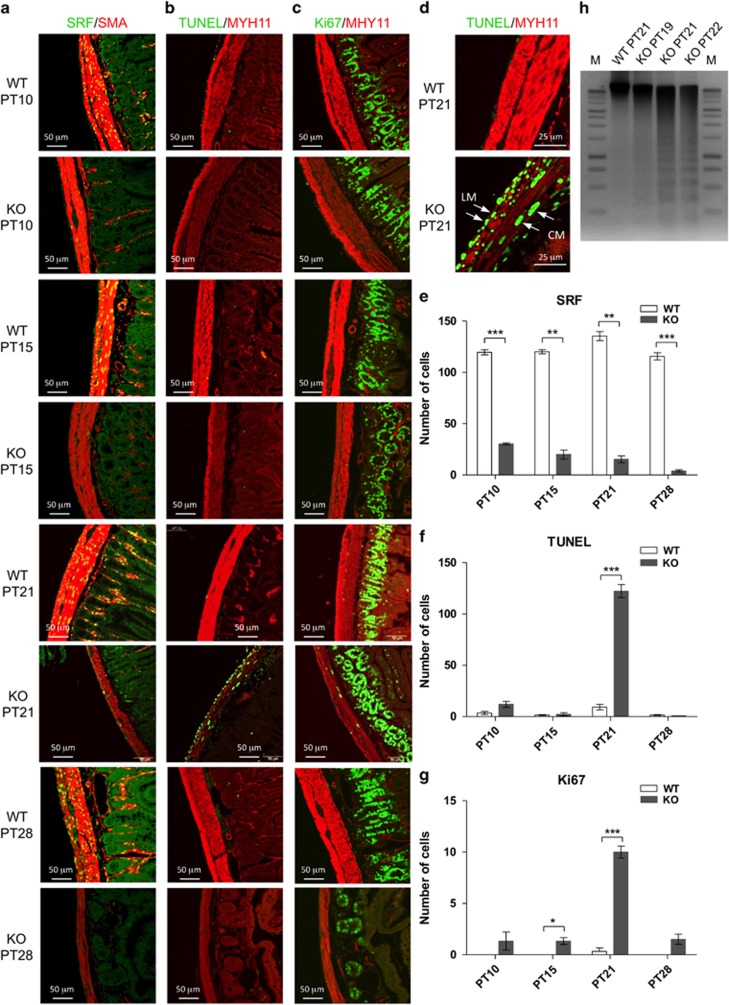
Increased apoptotic and proliferative activities as SRF depleted in *Srf* KO SMCs. (**a**) Cryosection images of jejunum showing gradual depletion of SRF (green, nucleus) and reduction of SMA (red, cytoplasm) in KO SMCs compared with WT control at 10, 15, 21 and 28 days post-tamoxifen injection (PT10–PT28). (**b**) Cryosection images of PT10–PT28 showing reactivity of TUNEL (green, nucleus), which was significantly increased in KO SMCs at PT21, compared with WT control. Expression of MYH11 (red, cytoplasm) was significantly less in apoptotic SMCs. (**c**) Cryosection images of PT10–PT28 showing reactivity of Ki-67 (green, nucleus), which was slightly increased in KO SMCs at PT21, compared with WT control. Expression of MYH11 (red, cytoplasm) was significantly less in KO SMCs. (**d**) High-magnification images of TUNEL reactivity in longitudinal (LM) and circular muscle (CM) layers at PT21 of KO and WT. Quantification of SRF^+^ cells (**e**), apoptotic (TUNEL^+^) cells (**f**) and proliferating (Ki-67^+^) cells (**g**) in the jejunal muscle layers of KO and WT in cryosection images (**a**–**c**) of PT10–PT28 (each *n*=3). Values are expressed as means±S.E. *P<0.01, **P<0.001, ***P<0.0001 *versus* WT. (**h**) Analysis of apoptotic DNA. Genomic DNA (500 ng of each) isolated from PT19, PT21 and PT22 of WT and/or KO was analyzed on a 1% agarose gel with a 10-kb DNA ladder

Time-lapsed examination of jejunal smooth muscle following tamoxifen-induced *Srf* KO revealed that SRF protein expression was significantly decreased in the longitudinal and circular muscle layers by PT10 and nearly undetectable by PT28 (Figure 3a). The number of SMCs expressing SRF in KO muscle layers also decreased significantly and gradually with the progressive time points compared with WT SMCs, whose SRF levels remained unchanged (Figure 3e). Noticeable degeneration of smooth muscle (as labeled with anti-SMA (ACTA2) and anti-MYH11 in Figure 3a–c), however, did not occur until PT21 and PT28.

Interestingly, terminal deoxynucleotidyl transferase dUTP nick-end labeling (TUNEL) signal was detected in *Srf* KO SMCs at PT21, which indicated that the degenerative changes were at least partially mediated by apoptosis (Figures 3b and d). Of note, there was little or no TUNEL signal in WT and KO SMCs at PT10, PT15 and PT28 suggesting that apoptotic degeneration was essentially complete by 3 weeks after tamoxifen injection (Figure 3b). Furthermore, the average number of apoptotic cells detected in KO muscle layers at PT21 (Figure 3b) was 122.3 (Figure 3f), whereas the average number of SRF^+^ SMCs in WT muscle layers at PT21 (Figure 3a) was 135.3 (Figure 3e). The relatively close numbers of SRF^+^ SMCs and apoptotic cells at PT21 indicated that most SMCs had undergone apoptosis. Finally, we detected DNA fragmentation, a hallmark of apoptotic cell death, starting at KO PT21 and progressing to KO PT22. However, no DNA fragmentation was observed in WT PT21 and KO PT19 (Figure 3h).

Along with apoptosis induction, there was a slight increase in cellular proliferation in the *Srf* KO SMCs of both muscle layers as revealed by anti-Ki67 staining (Figure 3c). More specifically, SMCs of *Srf* KO mice exhibited increased proliferative activity at PT21 but not at PT10 and PT15 when compared with WT (Figure 3c). Overall, there were more proliferative cells in the muscle layers of KO than WT mice at the specified time points; particularly, there was a significant increase in cellular proliferation at KO PT21 (Figure 3g). Notably, there was relatively high proliferative activity in the mucosal crypts, serosa and MY region at all-time points. Furthermore, there were significantly less cryptic epithelial cells in the KO mucosa suggesting that cryptic stem cells may need the supportive environment of SMCs for growth and survival (Figure 3c). Altogether, our data indicated that in the absence of SRF, SMCs lost their contractile phenotype and underwent significant apoptosis at PT21 with a transient increase in proliferative activity possibly as a compensatory response for the loss of functional SMCs.

### Genetic changes of SRF-induced miRNAs in KO SMCs

To examine the effects of SRF KO on miRNA expression in SMCs, we sequenced the miRNAs expressed within the muscle layers of the more dilated upper and less dilated lower regions of the jejunum in WT and *Srf* KO mice at PT15 (Figure 1b). Of the approximately 50 million raw sequence reads that were acquired from these four samples, 44.8 million reads were mapped to the mouse genome, which led to the identification of 9–11 million annotated miRNAs from each sample (Table 1). The annotated miRNAs revealed more miRNA species present in the *Srf* KO tissue than that of WT: 806 in KO upper jejunum *versus* 694 in WT upper jejunum; 750 in KO lower jejunum *versus* 644 in WT lower jejunum. A full list of annotated miRNAs with expression levels is available in Supplementary Table 1.

**Figure 4:**
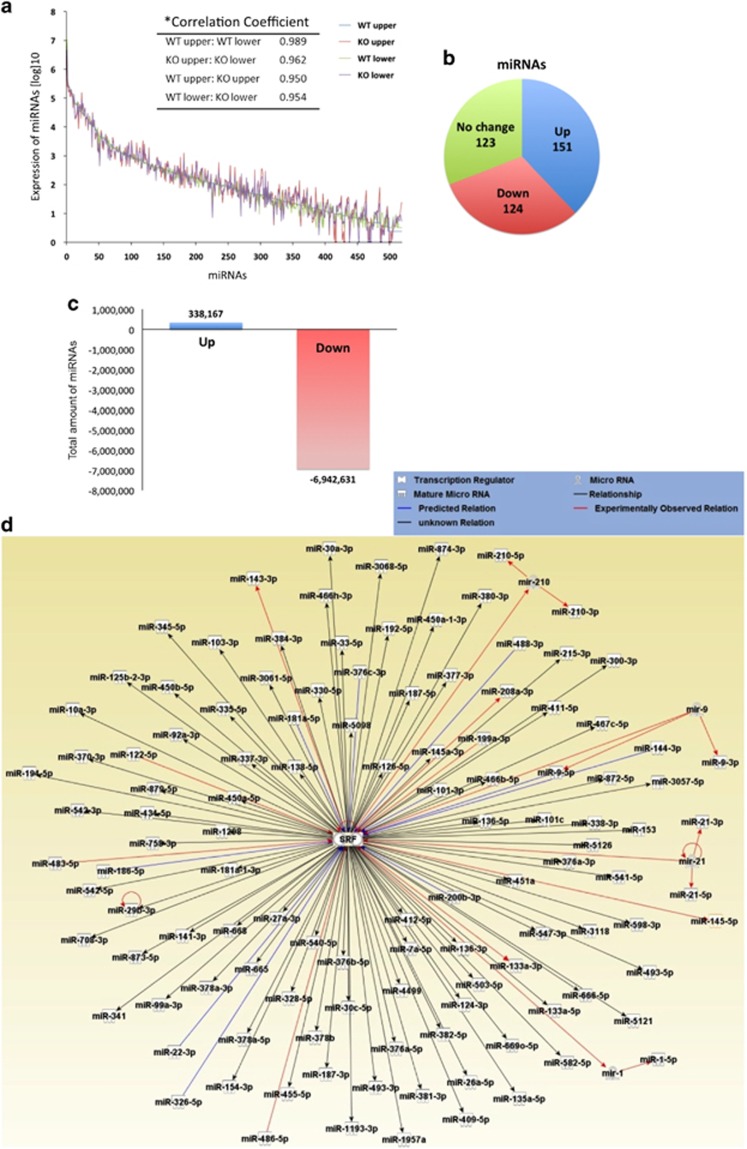
Identification of dysregulated miRNAs in the KO smooth muscle. MiRNA-seq analysis of KO upper and lower jejunum along with WT controls identified 892 miRNAs. After filtering by expression levels, 519 miRNAs were used for further analysis. (**a**) Expression profile of miRNAs identified in WT and KO smooth muscle. (**b**) Number of dysregulated miRNAs. (**c**) Comparison of total dysregulated miRNAs between WT and KO. (**d**) Pathway analysis of SRF-dependent miRNAs. Interactions between upregulated miRNAs and SRF were analyzed using IPA software

Interestingly, there was a high correlation coefficient (0.989) between the normalized miRNA expression profiles of WT upper and lower jejunum indicating a similar pattern of expression (Figure 4a). The miRNA expression patterns of KO upper and lower jejunum samples were also similar with a correlation coefficient of 0.962. However, the lower correlation coefficients between WT and KO upper jejunum miRNAs (0.950) and between WT and KO lower jejunum miRNAs (0.954) signified significant expression pattern changes in the KO samples.

After excluding minimally expressed miRNAs with <10 normalized reads, the expression profile analysis revealed that in *Srf* KO samples, approximately one-third of miRNA species (151; 37.9%) were upregulated and one-third (124; 31.2%) were downregulated (Figure 4b). When total amounts of miRNAs were considered, however, KO samples had significantly more downregulated miRNAs (6.9 million reads) than upregulated miRNAs (0.3 million reads) relative to WT samples (Figure 4c). The IPA software analysis showed that of the 124 miRNAs that were downregulated in *Srf* KO samples, 11 were known miRNAs (including SMC-specific miR-143 and miR-145), 8 were predicted miRNAs and 105 were unknown miRNAs that may have been directly or indirectly induced by SRF (Figure 4d). Thus, the *in vivo* miRNA data from *Srf* KO mice confirmed that SRF regulates the expression of most miRNAs predominantly expressed in jejunum SMCs (Supplementary Table 2) and that KO of *Srf* in SMCs results in a significant reduction in the expression levels of SRF-induced miRNAs.

### SRF-induced miRNAs target apoptotic proteins

**Figure 5:**
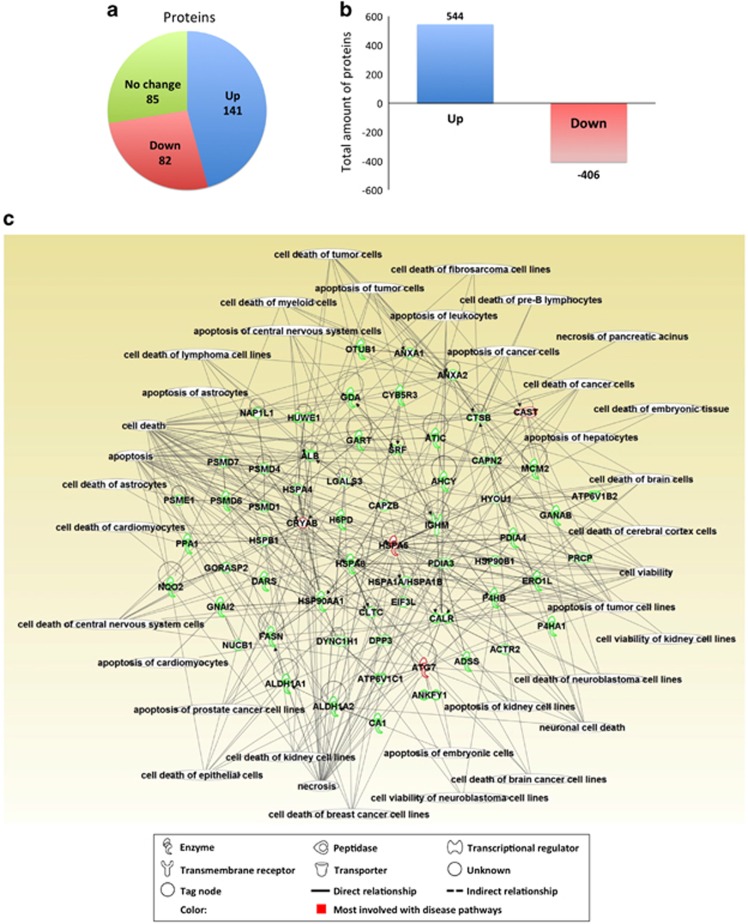
Identification of dysregulated proteins in the KO smooth muscle. LC/MS analysis of KO and WT jejunum (*n*=3 each) identified total 1531 proteins. The proteins were analyzed using Scaffold and filtered by probability (>95%) and *P*-value (<0.05) into 308 proteins, which were used for further analysis. (**a**) Number of dysregulated proteins in KO smooth muscle compared with WT controls. (**b**) Comparison of amount of dysregulated proteins between WT and KO. (**c**) Cell death pathway analysis of upregulated proteins. Relationships of upregulated proteins to cell death were analyzed using IPA

To identify the proteins modulated by SRF in GI smooth muscle, a proteomic analysis of *Srf* KO and WT jejunum smooth muscle at PT15 was performed using multidimensional protein identification technology. The analysis identified 1531 proteins, which were filtered by a statistical cutoff (95% probability) into 1035 proteins (Table 2). We anticipated a significant decrease in number of expressed proteins with the *Srf* KO because many smooth muscle contractile genes, including smooth muscle actin and myosin heavy chain, are direct targets of SRF.^1^ As expected, we found 82 downregulated proteins (Figure 5a), including several smooth muscle contractile proteins in *Srf* KO tissue (unpublished data). Interestingly, however, there were significantly more upregulated (141) than downregulated (82) proteins (Figure 5a), and overall, the total amount of upregulated proteins was greater than that of downregulated proteins (Figure 5b). This protein expression profile was consistent with loss in miRNA-mediated repression of target mRNA/proteins.

Based on our experimental results, we hypothesized that the downregulated proteins are directly associated with the early synthetic state phenotypic changes of SMCs, whereas the upregulated proteins are linked to the late degeneration of SMCs in *Srf* KO mice. The IPA software analysis of the upregulated proteins (Supplementary Table S3) in relation to cell death pathways revealed that most of these proteins were indeed linked to known apoptotic pathways in cells such as cardiomyocytes, neuronal cells, hematopoietic cells and cancer cells (Figure 5c). The identified apoptosis-associated proteins that may be responsible for SMC death in *Srf* KO mice included CAST, CRYAB, HSPA5 and ATG7.

**Figure 6:**
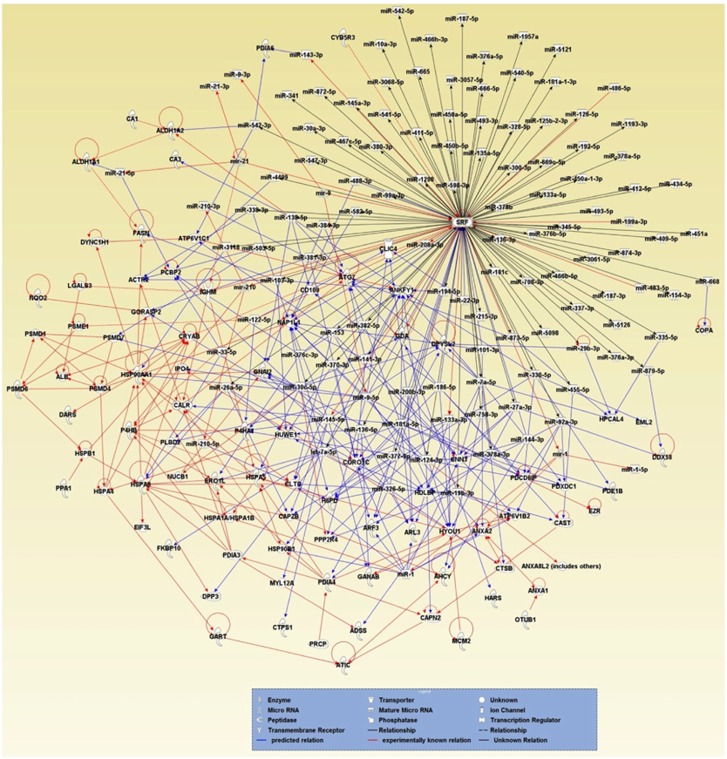
Interactions of SRF-dependent miRNAs and upregulated proteins. Interactions between SRF-dependent miRNAs and upregulated proteins were analyzed using IPA. Note that the SRF-dependent miRNAs target the upregulated proteins in *Srf* KO SMCs

Next, we tested the hypothesis that increased protein expression in *Srf* KO SMCs (Supplementary Table S3) is caused by downregulation of SRF-induced miRNAs (Figure 4d), which post-transcriptionally suppress the expression of these proteins. The IPA software analysis showed that most upregulated proteins were already known (red line) and/or predicted (blue line) targets of miRNAs induced by SRF (Figure 6). The investigation also revealed that SMC-specific miRNAs, such as miR-145-5p, miR-1 and miR-133a-3p, have multiple targets. IPA software generated targets for miR-145-5p included NAP1L1, PCB2, GORASP2, P4HA1, CAPZB, CTPS1, GANAB, HDLBP and DPYSL2; miR-1 targets included ANXA2, ATP6V1B2, CNN3, ARL3, CORO1C, ARF3, HSPA1A/B and HSP90B1; and miR-133a-3p targets include ARL3, HYUO1, PPP2R4 and CORO1C. Of these, eight targeted proteins (GORASP2, CAPZB, GANAB, ANXA2, ATP6V1B2, HSPA1A/B, HSP90B1 and HYUO1) were linked to cell death (Figure 5c). Thus, our proteomics data and pathway analyses suggested that SRF deficiency in KO SMCs causes aberrant expression of numerous apoptotic proteins by decreasing production of SRF-induced miRNAs that inhibit expression of the apoptotic proteins.

## Discussion

Although several animal studies have consistently demonstrated that SRF is a key regulator in the development and maintenance of both embryonic and adult muscle cells, SRF-deficient phenotypes in the heart and GI tract differ slightly depending on the gene promoters that are used for the expression of *Cre* recombinase. For example, congenital *Srf* gene mutageneses in mice result in severe defects in gastrulation (global KO),^14^ and heart development (cardiac muscle-specific *Myh6* promoter-driven KO^15^; vascular muscle-specific *SM22α* promoter-driven KO^16^) as well as in GI tract development (SMC-specific *Myh11* promoter-driven KO).^13^ Similarly, inducible *Srf* gene mutageneses in adult mice result in severe defects in the GI tract (*SM22α*^11, 12^ and *Myh11* promoter-driven KOs in this study). The *Myh6* and *SM22α* promoter-driven *Srf* KOs show a more severe effect on cardiac and/or smooth muscles than *Myh11* promoter-driven KO. For instance, the cardiac and/or GI defects manifest at around E12.5 (*Myh6*),^15^ E11.5 (*SM22α*)^16^ or E18 (*Myh11*)^13^ in congenital KOs, whereas the GI defect manifests at around 13–14 (*SM22α*),^11, 12^ or 21 days (*Myh11* in this study) after tamoxifen injection in the inducible KOs. This phenotypic variation may be explained by the time of activation and strength of the promoters. For example, *SM22α-Cre* activates earlier at E9.5 (ref.^17^) relative to *Myh11-Cre*, which begins to express at E13.5,^18^ and *SM22α (Tagln)* has higher levels of expression than *Myh11* in jejunal and colon SMCs.^19^ Furthermore, *Myh11* is expressed only in differentiated SMCs,^20^ whereas *SM22α* is expressed in both proliferating and differentiated SMCs.^21^ Although proliferating SMCs may represent mature SMC precursors, such as myofibroblasts, these cells are not true SMCs phenotypically. Therefore, the phenotypes from the congenital and inducible *Myh11-Cre*-driven *Srf* KO mice shown in this study more precisely reflect the role of SRF in embryonic and adult SMCs.

We previously reported that GI smooth muscle of mice expressed 312 miRNAs, of which 36 were SRF dependent as evidenced by *in vitro* Srf knock-down.^10^ Using miRNA-seq analysis, we have presented here more comprehensive miRNA profile of the GI smooth muscle from an *in vivo Srf* KO mouse model. The genome-scale miRNA profile obtained from this model consisted of 891 miRNAs, of which 124 were induced by SRF. Our results were further validated by the finding that 12 of the 36 SRF-dependent miRNAs, including miR-143 and miR-145 that were discovered through *in vitro* Srf knock-down were also found to be SRF dependent in the *in vivo Srf* KO model. Surprisingly, over 95% of miRNAs expressed in SMCs were affected by SRF in our *in vivo* model further alluding to the complexity of SRF influence on epigenetic regulation.

Among the miRNAs predominately expressed in SMCs, miR-143 and miR-145 are the most well established, and not surprisingly, these two miRNAs account for 78% of all miRNAs expressed in the smooth muscle of our *in vivo Srf* KO model (Supplementary Table 1). This finding can be partly explained by the fact that miR-143 and miR-145 are generated from the same primary transcript, and binding of SRF to a conserved CArG box located in the distal promoter region modulates their expression.^22^ As SRF-induced miR-143 and miR-145 expression promotes GI SMC differentiation and suppression of proliferation,^10^ deficiency of *Dicer*, which prevents generation of mature miRNAs, can lead to degeneration of SMCs in GI smooth muscle.^9^ This SMC degeneration resembles that of *Srf* deficiency suggesting that both SRF and Dicer may be on the same regulatory pathway of SMC differentiation and survival.

Collectively, our data indicate that SRF-induced miRNAs have a critical role in SMC apoptosis and offers an explanation for why SMCs could not survive without miRNAs in the *Dicer* deficiency model.^9^ Mechanistically, our findings suggest that SRF is the major regulator of predominantly expressed miRNAs in SMCs, which account for 95% of miRNAs expressed in SMCs. This includes miR-143/145 that function to target and inhibit apoptotic proteins (Figure 6).

Remarkably, 63 of 141 proteins that were overexpressed with loss of SRF-induced miRNAs were associated with the massive apoptotic activity in *Srf* KO muscle at PT21 (late stage). The cell death occurred without necrotic disruption of plasma membrane (Figure 2b), which was consistent with apoptosis. DNA fragmentation, which is a hallmark of apoptosis, was detected in our studies (Figure 3h). Apoptosis naturally occurs in cells during development and aging, and apoptotic bodies are immediately phagocytosed by macrophages or adjacent normal cells.^23^ SMC apoptosis in *Srf* KO muscle occurs in a narrow time window around PT21–P22 (Figure 3). The process of phagocytosis or removal of apoptotic bodies follows instantly to avoid an inflammatory immune response.^23^ The presence of inflammation in the smooth muscle as early as PT15–P18 suggests that there may have been SRF-independent changes before the induction of apoptosis. Our data, therefore, provides evidence that loss of SRF leads to apoptosis through loss of inhibitory miRNAs and offers insight into a new mechanism for SMC degeneration, which may occur under certain pathophysiological conditions.

Although the role of SRF in SMC growth, differentiation and phenotypic maintenance has been well established,^1, 2, 3^ the evidence of SRF in apoptosis has only recently begun to emerge. Parlakian *et al.*^24^ first observed that the number of apoptotic cells increase within the embryonic heart of the cardiac muscle-restricted (*Myh7*) *Srf* KO mice. Next, using a mammary epithelial cell model, Wiese *et al.*^25^ showed that restoration of SRF antagonizes Myc repression of SRF target genes, attenuates Myc-induced apoptosis, and reverts a Myc-dependent decrease in Akt phosphorylation and activity. The authors also noted that there was no evidence for direct interaction of Myc or Miz1 with SRF and that Myc/Miz1 complexes most likely repressed SRF target genes by recruiting histone deacetylases to joint target promoters because transcriptional activation by SRF requires histone acetylation.^25^ Furthermore, Sisson *et al.^6^* also recently demonstrated that the *in vivo* administration of CCG-203971, a SRF/MYOCD-related transcription factor (MRTF) pathway inhibitor, into two mouse lung fibrosis models promotes myofibroblast apoptosis, decreases alveolar plasminogen activator inhibitor-1 and leads to significantly reduced lung collagen content, thereby decreasing lung fibrosis. In another study examining the role of SRF in hepatocellular carcinoma resistance to sorafenib, an oral multi-kinase inhibitor, Bae *et al.^5^* showed that antisense inhibition of SRF expression in SH-J1 cells significantly enhances the apoptotic effects of sorafenib while reducing expression of mesenchymal markers and restoring expression of E-cadherin. In regards to miRNA involvement in apoptosis, Chen *et al.*^26^ also recently reported their discovery of SRF as a potential target for miR-320a through bioinformatics analysis, which was validated by *in vitro* and *in vivo* studies. In the latter, the authors noted that miR-320a contributes to atherogenesis by downregulating SRF, inhibiting human-derived endothelium cell proliferation and inducing apoptosis.^26^ Consistent with their findings, we also found that miR-320a was increased twofold in our miRNA-seq analysis of the *Srf* KO GI smooth muscle compared with WT control (Supplementary Table 1).

Interestingly, our ultrastructural and TUNEL assay findings in the GI smooth muscle of *Srf* KO mice were strikingly similar to that of the aortic smooth muscle in SMC-specific *Myocd* (myocardin) KO mice generated by Huang *et al.*^7^ Mirroring our results, Huang *et al.^7^* noted increased TUNEL, caspase 3, caspase 9 and p53 activities at 4 months after tamoxifen exposure in their conditional KO of *Myocd* in SMCs. Consistent with programed cell death, the authors also noted nuclear chromatin aggregation, nuclear fragmentation and cytoplasmic apoptotic body formation on electron micrographs. Furthermore, the aorta of *Myocd* KO mice displayed autophagy markers at 14 days after tamoxifen exposure indicating that loss of MYOCD activated a stress-induced autophagy program. Grossly, Huang *et al.^7^* also noted significant dilation of the stomach, small intestine, bladder and ureters in the *Myocd* KO mice at 4 months following tamoxifen administration. The remarkable similarities in the gross, microscopic and molecular findings of their *Myocd* KO and our *Srf* KO mice models are not surprising given that MYOCD is an important MTRF transcriptional coactivator that binds directly to SRF to activate synergistically the transcription of a subset of SRF-regulated genes that encode cytoskeletal and contractile proteins.^3, 27^ Other MRTFs include MRTF-A/MKL1/MAL and MRTF-B/MKL2,^3^ and interestingly, MRTF-B/MKL2 KO mice have a phenotype that is different from that of mice lacking MYOCD, which underscores their distinctive roles in SMCs.^28^ Importantly, the combination of our results and that of Huang *et al.* indicates that both SRF transcription factor and the MYOCD cofactor are equally necessary for the development and maintenance of the contractile phenotype, as well as suppression of apoptotic activity in GI SMCs.

In absence of SRF, the GI smooth muscle temporarily hypertrophied at PT15 (Figure 1d). This transient growth was supported by evidence of increased numbers of proliferating cells in the muscle layers at PT10 and PT15 (Figure 3g). We recently found that SMCs dedifferentiate into myofibroblast-like cells that express low levels of PDGFR*α* (PDGFR*α*^low^ cells) and SRF in a partial bowel obstruction injury model.^13^ The PDGFR*α*^low^ cells represent actively proliferating cells that are responsible for the hypertrophic response of the smooth muscle.^13^ In the SRF-deficient mice of this study, SMCs may transform into these proliferative PDGFR*α*^low^ cells during the early period following SRF ablation. The rapid and global loss of SRF may later trigger apoptosis through the SRF-induced miRNA pathway, whereas the slow and gradual loss of SRF in SMCs that ensues with partial obstruction may enable dedifferentiation and continual growth of SMCs without apoptotic induction.

In conclusion, we propose that SRF has a new functional role in the suppression of apoptosis via SRF-dependent miRNAs in SMCs. Rapid ablation of SRF expression in *Srf* KO SMCs reduces expression of SRF-dependent miRNAs that in turn induce abnormal overexpression of apoptotic proteins of the miRNA targets. The latter ultimately leads to SMC death. These findings provide new insight into how pathophysiological conditions of SMC degeneration may develop within the GI tract.

## Materials and Methods

### Generation of inducible *Srf* KO mice

The SMC-specific inducible *Srf* KO mouse line *Tg(Myh11-Cre-ERT2);Srf*^*lox/lox*^ was generated by cross-breeding a *Srf*^lox/lox^ female homozygote mouse (The Jackson Laboratory, Bar Harbor, ME, USA) with *Tg(Myh11-Cre-ERT2)* male mouse^29^ according to procedures approved by the Institutional Animal Care and Use Committee at the University of Nevada, Reno.

### Tissue preparation

The transgenic KO and WT mice were anesthetized by isoflurane inhalation after multiple time points following tamoxifen injection. Stomach, small intestine and colon were dissected from the mice. Whole tissue was used for hematoxylin and eosin (H&E) staining, electron microscopic examination, immunohistochemistry, proliferation and apoptosis assays. Smooth muscle stripped free of mucosa and submucous plexus was used for RNA-seq, DNA fragmentation analysis, proteomics and confocal microscopic examination.

### miRNA-seq

Small RNA libraries were generated using the Illumina TruSeq Small RNA Preparation Kit (Illunima, San Diego, CA, USA) according to the manufacturer's instructions. The cDNA libraries were sequenced via Illumina GAIIx (Illunima, San Diego, CA, USA) following the vendor's instruction at LC Sciences (Houston, TX, USA). Sequence reads were extracted from the image files using Illumina's Sequencing Control Studio software version 2.8 (SCS v2.8) following real-time sequencing image analysis and base-calling by Illumina's Real-Time Analysis version 1.8.70. A proprietary pipeline script, ACGT101-miR v4.2 (LC Sciences), was used for sequencing data analysis. All miRNAs were annotated from pre-miRNA and mature miRNA sequences listed in the latest release of miRBase.^30^ miRNA expression profiles for each sample were generated for comparison by normalizing with the total number of miRNAs.

### DNA fragmentation analysis

Genomic DNA was extracted from jejunal smooth muscle with AllPrep DNA/RNA/Protein Mini Kit (Qiagen, Hilden, Germany) according to the manufacturer's instructions. DNA electrophoresis was carried out on 1% agarose gel in TAE buffer at 50 V for 2 h. DNA was stained with ethidium bromide before viewing and imaging by GelDoc XR (Bio-Rad Laboratories, Milan, Italy).

### Proteomics analysis

Soluble proteins were prepared from jejunum tissues of WT and *Srf* KO mice (*n*=6). Each extract contained proteins from two mice. Proteins in the extracts were trypsin digested and analyzed by two-dimensional LC-MS/MS on a Thermo Finnigan LTQ-Orbitrap (Thermo Scientific, Fremont, CA, USA). Data were analyzed using Sequest (The Scripps Research Institute, La Jolla, CA, USA) and validated with Scaffold (Proteome Software, Portland, OR, USA). Proteins were quantified by spectral counting and potentially perturbed molecular pathways were found with Ingenuity Computational Pathway Analysis (IPA; QIAGEN, Redwood City, CA, USA).

### Bioinformatics analysis

Genes of miRNAs and mRNAs regulated by SRF were analyzed using IPA. The IPA software analyzed relationships (1) among downregulated miRNAs and SRF (Figure 4d), (2) upregulated proteins and cell death or apoptosis (Figure 5c) and (3) among downregulated miRNAs, upregulated proteins and SRF (Figure 6). Genomic location of exons, CArG boxes (collectively termed the 'CArGome') and SRF-binding sites (ChIP data available in UCSC ENCODE) in the genes of transcriptional variants were analyzed using Smooth Muscle Cell Genome Browser,^19^ which was custom-built on the UCSC Genome Browser.^31^ The CArGome Search Browser within the Smooth Muscle Cell Genome Browser was updated with the transcriptome data of primary SMCs isolated from colon and jejunum.^19^

### Confocal microscopy and histological analysis

Jejunum and colon tissues were analyzed by whole mount and cryostat section staining using confocal microscopy as previously described.^32^ Cell-specific primary antibodies against the following antigens were used: anti-MYH11 (1 : 800, Alfa Aesar, Ward Hill, MA, USA) for SMCs, CD117/c-kit (1 : 20, R&D Systems, Minneapolis, MN, USA) for ICCs or PGP9.5 (1 : 1000, UltraClone Limited, Wellow, UK) for enteric nerve system. For histological analysis, jejunum tissues were dehydrated, embedded in paraffin, cut into 4 *μ*m-thick coronal sections, rehydrated and stained with H&E. Images were collected using an Olympus FV1000 confocal laser scanning microscope with Fluoview FV10-ASW 3.1 Viewer software (Olympus, Tokyo, Japan) or the iScan Coreo scanner (Ventana Medical Systems, Tucson, AZ, USA).

### Proliferation and apoptosis assays

Whole jejunums were fixed for proliferating or TUNEL (TdT-mediated X-dUTP nicked labeling) assays. The proliferation assay was performed with PFA-fixed sections double stained with anti-Ki-67 (1 : 1, BioLegend, San Diego, CA, USA) and anti-MYH11 (1:800, Alfa Aesar) overnight at 4C°. Apoptotic assay was performed both using TUNEL reaction (Roche, Indianapolis, IN, USA) and double stained with anti-MYH11. Tissues were also double stained with anti-SRF (1:500, Santa Cruz Biotechnology, Dallas, TX, USA) and anti-*α*SMA (1:1200, Sigma, St. Louis, MO, USA). Stained tissues were analyzed using confocal microscopy.

### Electron microscopy

Whole jejunum and colon tissues were fixed in a fresh fixation buffer (3% glutaraldehyde and 4% paraformaldehyde in 0.1 M phosphate buffer, pH 7.4) for several days at room temperature. Specimens were then post-fixed in 1% osmium tetroxide for 2 h at 4 °C, rinsed in distilled water, block stained with saturated aqueous uranyl acetate solution for 3 h, dehydrated in a graded series of ethyl alcohol and embedded in EPON 812. Ultrathin sections were stained with uranyl acetate and lead citrate and examined under microscope (Hitachi H-7650, Tokyo, Japan).

### Statistical analysis

Protein quantification was done by spectral counting and the data were analyzed using the in program statistical package provided with Scaffold. Scaffold was used to generate *P*-values for proteins based on quantitative information in biological groups and replicates. Proteins were filtered by probability (>95%) and *P*-value (<0.05) for further analysis. The statistical significance level for three-way comparisons of spectral counts measures was pre-determined as 0.05. In the quantification of SRF^+^, apoptotic and proliferating cells, the two tailed *t*-test was used to compare cell numbers, and differences with *P*-values of <0.05, <0.001 and <0.0001 were considered statistically significant.

## Figures and Tables

**Table 1:** Summary of miRNA sequencing data

**Sample**	**Raw reads**	**Mapped**	**miRNAs**	
			**Number^a^**	**Species^b^**
WT upper jejunum	13 789 348	12 464 495	10 847 500	694
KO upper jejunum	12 993 528	11 328 659	9 635 255	806
WT lower jejunum	11 599 001	10 576 282	9 132 527	644
KO lower jejunum	11 793 310	10 457 773	9 067 347	750

a Total anotated reads of miRNAs.

b Total species of miRNAs.

**Table 2:** Summary of proteomics study

**Sample**	**Total^a^**	**Trimed^b^**	**Number of protein**		
			**Upregulated^c^**	**Downregulated^d^**	**No change^e^**
WT upper jejunum					
KO upper jejunum	1531	1035	141	82	85
WT lower jejunum					
KO lower jejunum					

a Total proteins identified from the two-dimensional LC/MS/MS.

b Proteins filtered by probability (>95%).

c Fold changes are >2.0.

d Fold changes are <0.5.

e Fold changes is equal to 1.0.

## Abbreviations

SRF: serum response factor
MYOCD: myocardin
SMCs: smooth muscle cells
miRNAs: microRNAs
CArG box: [CC (A/T)_6_ GG]
CArGome: genome-wide CArG boxes
MRTFs: myocardin-related transcription factors
KO: knockout
WT: wild type
GI: gastrointestinal
PT: post-tamoxifen
ICC: interstitial cells of Cajal
MY: myenteric
DMP: deep muscular plexus
MLC: macrophage-like cells
TUNEL: terminal deoxynucleotidyl transferase dUTP nick-end labeling
H&E: hematoxylin and eosin
